# Dairy and Plant-Based Milks: Implications for Nutrition and Planetary Health

**DOI:** 10.1007/s40572-023-00400-z

**Published:** 2023-06-10

**Authors:** Rebecca Ramsing, Raychel Santo, Brent F. Kim, Daphene Altema-Johnson, Alyssa Wooden, Kenjin B. Chang, Richard D. Semba, David C. Love

**Affiliations:** 1 Johns Hopkins Center for a Livable Future, Johns Hopkins University, Baltimore, MD USA; 2 Department of Environmental Health and Engineering, Johns Hopkins Bloomberg School of Public Health, Baltimore, MD USA; 3 Department of Human Centered Design, College of Human Ecology, Cornell University, Ithaca, NY USA; 4 Wilmer Eye Institute, Johns Hopkins University School of Medicine, Baltimore, MD USA

**Keywords:** Dairy, Dairy alternatives, Health, Nutrition, Environment, Retail

## Abstract

**Purpose of Review:**

Dairy milk products are dominant in the market; however, plant-based milks are gaining prominence among USA consumers. Many questions remain about how plant-based milk products compare to dairy milk from a nutrition, public health, and planetary health perspective. Here, we compare the retail sales, nutrient profiles, and known health and environmental impacts of the production and consumption of dairy and plant-based milks and identify knowledge gaps for future studies. For our plant-based milk comparisons, we reviewed almond, soy, oat, coconut, rice, pea, cashew, and other plant-based milks as data were available.

**Recent Findings:**

The retail unit price of plant-based milks was generally higher than that of cow’s milk, making it less accessible to lower-income groups. Many plant-based milks are fortified to match the micronutrient profile of dairy milk more closely. Notable differences remained, especially in protein, zinc, and potassium, depending on the base ingredient and individual product. Some plant-based milks contain added sugar to improve flavor. Plant-based milks were generally associated with lower environmental impacts (e.g., greenhouse gas emissions, water use) than cow’s milk, with the notable exception of the higher water footprint of almond milk.

**Summary:**

This review of recent studies and consumer purchases confirmed that retail sales of plant-based milks are increasing and shifting among products. Further research is needed to better characterize the environmental impacts of newer plant-based milks, such as cashew, hemp, and pea milks; consumer attitudes and behavior towards plant-based milks; and the safety and potential health effects related to their long-term and more frequent consumption.

**Supplementary Information:**

The online version contains supplementary material available at 10.1007/s40572-023-00400-z.

## Introduction

Consumption of dairy (foods containing or made from animal milk) in the USA has shifted over the past few decades, with important implications for both human nutrition and planetary health. Fluid milk makes up the largest component of dairy consumption by weight; however, consumer demand has declined since the mid-1940s, with the largest relative decrease over the last decade [1]. This drop is accompanied by a growing demand for cheese and yogurt and a rapid increase in the demand for plant-based alternatives—often referred to as plant-based milks, cheeses, yogurts, and butters, typically made from soy, nuts, legumes, seeds, and grains [2]. The shift in dairy consumption has been attributed to changing demographics, food environments, consumer preferences, and labeling policies [3]. Plant-based milk sales volume increased 20% in 2020, with annual revenue growth twice the rate of dairy milk [4•]. Plant-based milks now make up 15% of retail dollar sales of milk, and an estimated 40% of households purchased plant-based milks in 2020 [4•]. As the US dairy alternative market continues to expand, diversify, and grow, there is a growing interest in the impacts that these changing consumer behaviors will have on the quality and nutritional content of individuals’ diets and the associated environmental impacts.

## Approach

The focus of this paper is dairy (cow) and plant-based milk consumption in the USA. We conducted a non-systematic review of the literature published from 2015 to 2022 using PubMed. We searched for articles pertaining to health and environmental impacts of dairy and plant-based milks (dairy alternatives) as well as market and consumer trends for these products.

We collected data on nutritional properties of dairy and non-flavored plant-based milks using the Food and Nutrient Database for Dietary Studies for 2017 to 2018 [5] and retail price data for liquid milk and plant-based milks from Nielsen (eXtended All Outlet Combined, xAOC, product, New York, NY) for years 2017 to 2019 using methods described previously [6, 7]. Retail outlet types included in this dataset were grocery market, chain supermarket, big box and club stores, Walmart, and military commissary.

We compiled literature on the greenhouse gas emissions (GHGe) and water use associated with the production of cow’s and plant-based milks. For GHGe, wherever possible, we standardized the scope of supply chain stages to span farm to processor gate, including packaging. Some studies included relatively small GHGe contributions from post-processor gate activities that we could not disaggregate. For water use, scope and metrics varied widely, so rather than attempting to standardize across studies, we compared plant-based milks to cow’s milk within each study. See the Supplementary Information for more details on the search strategy and metric standardization for GHGe and water use analysis.

## Background on Plant-Based Milks in the USA

Plant-based or non-dairy alternatives are designed to mimic the texture and qualities of dairy products, most often milk but also cheese, yogurt, ice cream, and butter. Though liquid dairy alternatives have traditionally been labeled as milks, such as soymilk or rice milk, more recently there has been legal debate regarding the use of these terms, with some industry stakeholders calling for the Food and Drug Administration to reserve the term “milk” solely for liquid secreted from animals [2].

Plant-based milks made from soy, rice, and coconut have been consumed and used in recipes for centuries but did not become widely available to American consumers until the early twentieth century [8]. Soymilk was first manufactured in the USA in 1917, where it was used largely as a substitute for cow’s milk in infant formula [9]. A 1995 study later found that consumption of soy protein was associated with a decrease in total cholesterol, LDL cholesterol and triglycerides [10], prompting more widespread consumption. In 1996, WhiteWave Foods started marketing soymilk in the refrigerated aisle alongside regular dairy milk. Sales increased throughout the late 1990s and early 2000s but slowed when new research suggested that soy may be an endocrine disruptor, due to its isoflavone molecules bearing a structural similarity to estrogen [9]. Subsequent research has concluded that consumption of soy does not cause any long-term harmful effects, though the American Academy of Pediatrics recommend against the exclusive use of soy formula for infants in most cases [11]. In 2008, the same year soymilk sales peaked, almond milk was first sold in refrigerated cartons by Blue Diamond [12]. A growing interest in veganism and plant-based eating resulted in a continued interest in almond milk. Almond milk sales overtook soymilk in 2014, and almond milk is currently the highest-selling plant-based milk in the USA [13]. A variety of milks derived from other plants have entered the market in recent decades, such as oat milk, which was first sold in the USA by Pacific Foods in 1996. The Swedish company Oatly, which entered the US market in 2016, is responsible for the dramatic growth of oat milk over the past few years due to a strategic marketing campaign [14]. Sales have more than doubled since 2017 and surpassed soymilk in 2021 but are still well behind almond milk [13]. Other products introduced in recent decades include cashew, hemp and pea milk, and those that combine multiple plants into one milk.

**Fig. 1:**
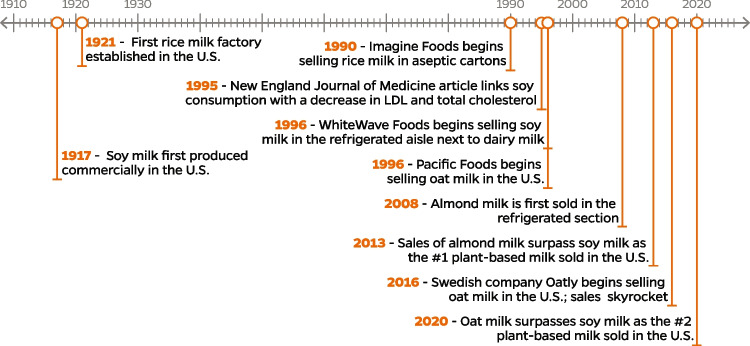
Timeline of plant-based milks in the USA

The timeline below (Fig. 1) describes the major shifts in the US plant-based milk market over the past century.

## Consumer Motivations for Shifts in Dairy Consumption

Digestibility and lactose intolerance, or the malabsorption of the lactase enzyme, are primary reasons given by consumers for the uptake of plant-based milks [15]. Estimates of the prevalence of lactose intolerance vary depending on the assessment method used, but the overall prevalence is believed to be close to two-thirds (68%) of the global population (> 10 years of age) [16]. In the USA, an estimated 36% of people have lactose intolerance with higher prevalence among African Americans, American Indians, Asian Americans, and Hispanic/Latinos [17]. A smaller percentage of children (0.5 to 3% at age 1 year) have an allergy to cow’s milk, though this is often outgrown later in life [18].

Perceived healthfulness is another common reason for choosing dairy alternatives [19, 20]. Plant-based milks are typically lower in saturated fat and cholesterol than full-fat cow’s milk. Some studies have suggested that consuming cow’s milk is associated with cancer and all-cause mortality, but recent meta-analyses do not support this, especially with moderate dairy intake [21, 22]. Other concerns include exposure to contaminants in cow’s milk (i.e., hormones, pesticide residues, mycotoxins, metals, and antibiotics), particularly in conventionally produced milk [23]. Though not well established, dairy has also been associated with acne and dermatological problems [24].

Interest in sustainability as well as concern for the environment and animal welfare increasingly motivate consumers to purchase plant-based milks [15, 20, 25]. In a recent survey, 77% of consumers of dairy and plant-based milks agreed sustainability is important for the greater good. On the survey, purchasers of plant-based milks rated the importance of sustainability in the highest category, though many consumers demonstrated difficulty describing the contributing factors to sustainability [25, 26]. According to a 2022 survey by First Insight, Inc., Gen Z consumers are more willing to adjust their purchases and even pay more for products they perceive to be more sustainable, which has potential to further disrupt the market for plant-based milks [27].

## Retail Sales of Dairy and Plant-Based Milks

Nationally, dairy milk products have significantly higher sales volume and revenue than their plant-based counterparts (Table 1). On a volume basis, cow’s milk and plant-based milks made up 87% and 8% of total annual milk sales, respectively. By annual sales revenue, dairy milk sales were $24.9 B, compared to $2.2 B for plant-based milks. During our study period (2017–2019), dairy milk sales decreased by an average of $456 million/year, while the sale of alternative milks increased by an average of $123 million/year (Table S1).

**Table 1:** Sales of dairy and plant-based milks, Nielsen average of 2017–2019

Category	Annual quantity sold (million units)	% of category (units sold)	Annual sales (million dollars)	Unit price (dollars/unit)
**Total dairy milk products**	**26,577**	**100.0**	**24,867**	**2.73**
Cow’s milk	25,067	94.3	21,626	2.64
Lactose reduced/free milk	1,144	4.3	2,297	3.93
Egg nog	138	0.5	357	2.70
Buttermilk	169	0.6	287	2.18
Remaining milk	58	0.2	299	2.71
**Total plant-based milks (dairy alternatives)**	**2,139**	**100.0**	**2,221**	**3.17**
Almond milk	1,587	74.2	1,251	3.18
Soymilk	265	12.4	432	3.15
Coconut milk	100	4.7	195	2.81
Oat milk	22	1.0	54	3.17
Rice milk	42	2.0	80	3.29
Remaining plant-based milk	123	5.8	207	3.53

Among dairy milks, cow’s milk (specifically, whole, low fat, and nonfat milks) accounted for 94% of units sold, followed by lactose-reduced milks (4%), with remaining milks (egg nog, buttermilk, powdered, and flavored milks) making up less than 2% (Table 1, Table S2). The largest contributor to the decreases observed in the sales of dairy milk sales was the reduction in the sale of cow’s milk, which, on average, decreased by $691 million/year between 2017 and 2019. This aggregate reduction in dairy milk sales was partially offset by consistent increases ($217 million/year, on average) in the sale of lactose-reduced milk within this same period.

Among plant-based milks (Table 1, Table S2), almond and soymilk accounted for 74% and 12% of all annual units sold, respectively. Plant-based milk sales increased each year, driven predominantly by almond milk sales ($105 million/year). Soymilk sales decreased by an average of $49.8 million/year and oat milk sales increased by $63.5 million/year. From 2018 to 2019, oat milk sales increased by nearly 700%.

The unit price of plant milks was on average 20% higher than dairy milk, excluding lactose-free and other subcategories of milk. Lactose-free milk is the most expensive of the dairy milk categories—on average, it cost more than any of the plant-based milk alternatives represented in the Nielsen purchasing data and 150% that of dairy milk.

## Health Impacts of Dairy and Plant-Based Milks

Regular consumption of dairy appears to confer important health benefits for children and is beneficial or neutral for adults. Dairy is considered a good source for three of five nutrients of concern identified in the 2020–2025 US Dietary Guidelines: calcium, potassium, and vitamin D [28, 29]. The benefits most often associated with dairy consumption include decreased risk of fractures and improved bone mineral density [8], possibly associated with calcium, phosphorous, vitamin D, and protein. While there does not appear to be a clear relationship between dairy milk intake and fractures, a tendency toward lower rates of osteoporosis and hip fractures with moderate dairy intake has been observed [30]. Vitamin K in fermented dairy products may help bind calcium to bone proteins, enabling mineralization. One meta-analysis found an 18% reduction only in vertebral fractures with higher dairy intake, though greater effects were seen with yogurt and cheese compared to milk [31]. Furthermore, dietary patterns that incorporate dairy along with vegetables and fruit and lower red meat, such as the Mediterranean diet, have also been positively associated with bone health [32, 33,34•].

For risk of cardiovascular disease (CVD) and type 2 diabetes, studies have shown neutral or slightly favorable associations for various forms of dairy intake in USA and European contexts [35-37]. In a prospective cohort study across 21 countries, higher intake of dairy was associated with reduced risk of CVD and stroke, especially for lower dairy-consuming countries [38]. Yogurt and fermented milk specifically may play a particularly beneficial role due to favorable effects on immunity, inflammation, diarrhea prevention, and cardiovascular risk factors in clinical trials, possibly through modifying the gut microbiota [39].

Some discussion exists as to whether low-fat dairy products confer any benefit over whole-fat dairy, primarily concerning saturated fat content [35]. Recent findings actually demonstrate a somewhat lower disease risk (CVD, diabetes) with higher fat dairy consumption, particularly with fermented milk, yogurt, and cheeses [40]. These benefits may be more closely related to the complex matrices of dairy protein structures, the presence of probiotics, or the type of saturated fat, with a greater proportion of short- and medium-chain fatty acids and less palmitic and stearic acids than other animal proteins [41, 42].

In children and adolescents, milk plays a vital role as a source of protein, micronutrients, fatty acids, and growth factors important for growth and development. Studies consistently demonstrate benefits of milk and other animal-source food consumption for children’s growth, cognitive performance, and nutrition status. Dairy consumption is significantly associated with height and weight gain and bone mineral density in children [43, 44]. One study calculated the cost of certain nutrients of concern by food group. Milk and dairy products were the least expensive source of calcium and vitamin D and the second least expensive source of magnesium, potassium, and vitamin A [45].

On the other hand, healthy plant-based diets—diets with little or no animal products—are associated with lower risk of diabetes, CVD, some cancers, and healthier weights (body mass index) in adults [46]. Emerging research on the role of the microbiota may shed light on the potential for plant-based diets to modulate inflammatory markers and affect bone remodeling pathways, thus protecting bone health over the long term [47]. Some plant milks contain bioactive compounds, for example, isoflavones and phytosterols in soymilk, or antinutritional factors, such as phytates for oat milk [48, 49].

Although calcium is obtained largely through the intake of dairy products in the USA, with planning and a balanced diet, adequate levels of calcium can be obtained from plant-based foods, including dairy alternatives and other foods that are fortified with calcium and vitamin D. Clinical studies on plant-based diets and bone health are sparse, and randomized control trials and experimental studies are even rarer. Some small studies found higher bone turnover with plant-based diets that were also lower in calcium and vitamin D [50, 51]. A recent review of evidence concluded that plant-based diets are not harmful to adult bone health if calcium and vitamin D intake are adequate [52].

## Nutrient Differences Between Dairy and Plant-Based Milks

As mentioned above, dairy milk is a good source of protein, calcium, and many important micronutrients. The nutrition quality and taste of plant-based milks vary greatly [53]. As with dairy milk, plant-based milks are often fortified with vitamins D and A. Additionally, many plant-based products are also fortified with calcium, vitamin B12, and other micronutrients to better match the nutritional content of dairy milk. The overall protein, fat, and nutrient profile depend on the base ingredient. Fat content varies across both dairy and plant-based milks. Many plant-based milks, such as almond and cashew milk, are much lower in protein and other important nutrients for growth and wellness, such as zinc, potassium, and magnesium (Table 2). Additionally, plant-based milks often contain added sugars, flavors, or ingredients to improve taste and texture, thus affecting the overall health profile.

**Table 2:** Average nutrients in dairy and plant-based milks. [54] Source: US Department of Agriculture, Agricultural Research Service (2019)

Item, 240 mL (n, products)	Protein (g)	Calcium (mg)	Magnesium (mg)	Phosphorus (mg)	Potassium (mg)	Selenium (mcg)	Zinc (mg)	Choline (mg)	Riboflavin (mg)	Vitamin D (mcg)
**Daily Value**	**50**	**1,300**	**420**	**1,250**	**4,700**	**55**	**11**	**550**	**1.3**	**20**
Dairy milk	8.2*	309**	29	252**	387	4.8	1.1	44	0.33**	2.7*
Dairy milk, lactose free	8.2*	309**	29	252**	387	4.8	1.1	44	0.33**	2.7*
Almond milk	1.0	449**	15	22	163	0.2	0.1	8	0.02	2.4*
Cashew milk (2)	1.8	116	ND	ND	69	ND	ND	ND	ND	0.0
Coconut milk	0.5	459**	0	0	46	0.0	0.0	0	0.00	2.4*
Hemp milk (3)	2.3	204*	54*	199*	101	ND	0.6	ND	0.42**	2.5*
Oat milk (16)	2.7	248*	ND	170*	184	ND	1.0	ND	0.55**	2.3*
Pea milk (2)	7.5*	385**	0	ND	421	ND	ND	ND	ND	2.5*
Rice milk	0.7	288**	27	137*	66	5.4	0.3	5	0.35**	2.4*
Soy milk	6.1*	294**	33	176*	280	5.2	0.5	44	0.45**	2.7*

** meets 20% Daily Value, * meets 10% Daily Value, ND = no data available

We excluded plant milks that were sweetened, flavored, (e.g., vanilla, chocolate), or made from a blend of base ingredients. Dairy and soy milks reflect average of whole, low fat and fat-free products. Nutrient data for multiple products of the same milk type was averaged when standard reference not available; see Supplemental See Table S3 for absolute values

**Fig. 2:**
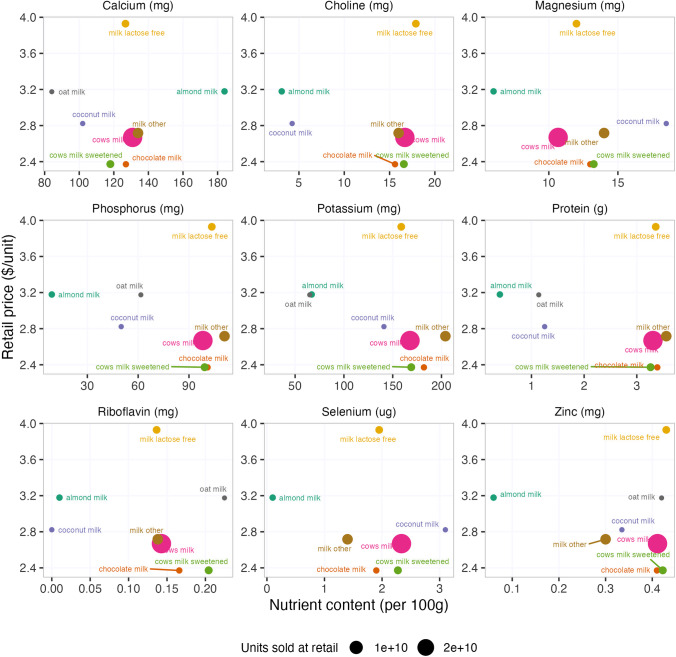
Average retail price ($/units) of milk and plant milks and their associated nutrient levels (per 100 g). Dot size represents the number of units sold at retail. Food and Nutrient Database for Dietary Studies for years 2017 to 2018 and retail price data for liquid milk and dairy alternatives from Nielsen 2017 to 2019. “Other milk” is goat milk

Figure 2 presents the average retail price ($/unit) of dairy and plant-based milks compared to their average nutrient value [28, 45]. Cow’s milk has a lower unit cost and higher nutrient value for protein as well as most micronutrients in the analysis, including choline, phosphorus, potassium, protein, riboflavin, and zinc. Almond and rice milk have the highest unit cost and lower values for protein. Oat milk is a more affordable source of zinc and riboflavin but is lower in protein, calcium, and potassium. While almonds are naturally rich in calcium, most almond beverage products are fortified with additional calcium. Notably, lactose-free milk has a similar nutrient profile as dairy milk, yet its unit cost is significantly higher across all nutrients.

## Environmental Impacts of Dairy and Plant-Based Milks

Dairy production is resource-intensive in terms of fresh water and land use and has an outsized contribution to global GHGe compared to producing plant-based foods [55, 56•, 57]. The rapid intensification and consolidation of dairy farms, particularly since the 1990s in the USA, has increased the efficiency of production, requiring less land and releasing fewer GHGe to produce the same output of milk [58]. However, intensification is associated with numerous other ecological, public health, and animal welfare concerns, including runoff and contamination of drinking water[58], antimicrobial resistance [59], and decreased biodiversity [58]. On the other hand, extensively grazing ruminant animals such as dairy cattle offers some benefits, such as creating human-edible protein from grazing land and fodder that would otherwise be unable to feed humans directly [60, 61] and biodiversity conservation in certain landscapes [62, 63].

**Fig. 3:**
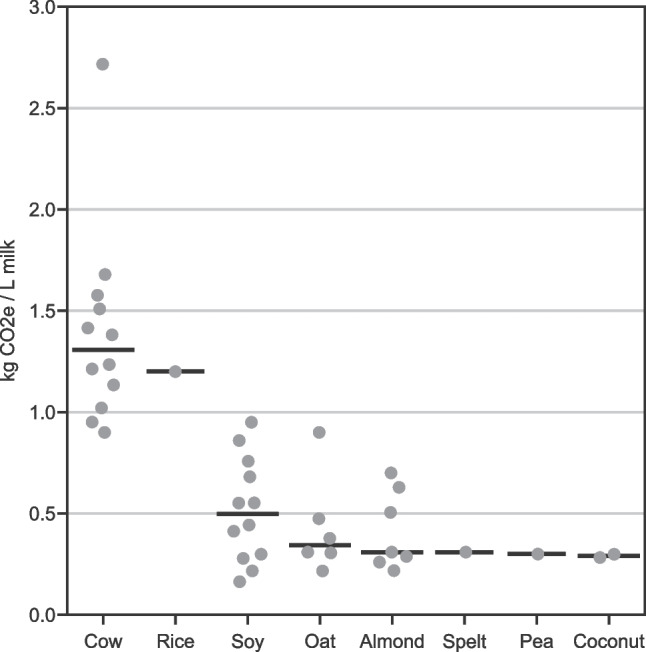
Greenhouse gas emissions (kg CO_2_e/L milk) associated with dairy and plant-based milks. Points represent single studies with a horizontal line at the median for each milk type. In cases where a single study reported multiple estimates (e.g., refrigerated and shelf stable; conventional and organic) for a given milk type, we averaged them to avoid over-representing results from those studies. See Tables S5–S7

Summaries of GHGe [64-67, 69-78,68••, ] and water use [66, 67, 71, 72, 75, 77, 79,68••, ] comparing plant-based milks to cow’s milk, compiled from life cycle assessment studies, are shown in Fig. 3 and Table 3 (see Table S4 for study details). Median per-liter GHGe (Fig. 3) associated with soy, oat, almond, spelt, pea, and coconut milks were 62–78% lower than that associated with cow’s milk. Among plant milks, rice was the most GHGe-intensive, although this is based on only one study.

**Table 3:**
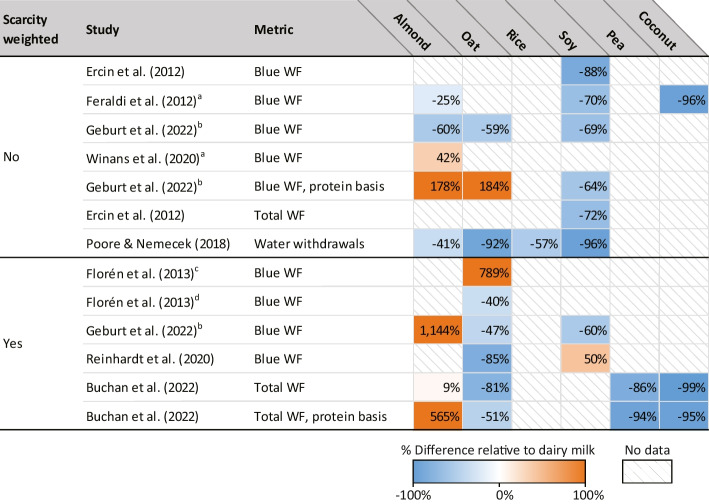
Water use for plant-based milks, relative to dairy milk

Percentages represent the magnitude of plant-based milk water use relative to dairy milk, e.g., in Ercin et al. [79], the estimated blue water footprint of soymilk was 88% lower than that of cow’s milk. Shading indicates whether plant milk water use was higher (orange) or lower (blue) compared to cow’s milk. Comparisons are made on a volume basis (e.g., per liter of milk) unless indicated otherwise (e.g., per gram of protein). See Supplemental Tables S8–S9 for absolute values

^a^Study did not include cow’s milk, so we compared plant milk(s) to the weighted global average cow’s milk estimate from Mekonnen and Hoekstra (2010)

^b^Average of conventional UHT, organic UHT, conventional non-UHT, and organic non-UHT cow’s milk; average of conventional and organic soymilk

^c^Cow’s milk from Sweden, oat milk from Germany

^d^Cow’s milk from Sweden, oat milk from Sweden

For comparative purposes, estimates shown in Fig. 3 were standardized, where possible, to span farm-to-processor gate GHGe. Emissions from post-processor gate activities (e.g., retail) were generally relatively small [64, 67, 71, 76], with the exception of one study [78]. This study found retail-related GHGe associated with refrigerated varieties of soy and almond milks to be an order of magnitude larger than those reported by other studies due to assumptions that these products have slower turnover than cow’s milk in stores and would require 3 to 5 days more refrigeration (hence more embodied energy than cow’s milk at retail). Further research is needed to clarify these substantial differences in retail-related emissions, especially given that the increasing popularity of plant-based milks may influence estimates about inventory turnover.

Comparing water use associated with producing plant-based milks is complicated by the wide range of metrics used in the literature. Studies in our review measured blue water footprint (WF), i.e., freshwater withdrawals from ground and surface sources lost to evaporation or incorporated into the product, such as for irrigation [66, 75, 79,68••, ]; total WF, a combined measure of blue, green (rainwater available to plants), and grey (polluted) water [77, 79]; and water withdrawals, which include all freshwater drawn from a source regardless of whether it returns to the watershed after use [71]. Some studies also weighted water use by the level of scarcity in the region from where it was withdrawn, for example, withdrawals from a water-scarce region such as California were weighted more heavily [67, 72, 77, 80,68••, ].

Across different water use metrics, per unit water use was often highest for almond milk, particularly when scarcity weighted or measured on a protein basis (Table 3). With a few exceptions, other plant-based milks were less water-intensive to produce than cow’s milk.

Other environmental impacts were less well represented in the literature. Geburt et al. [68••] found that oat milk scored as well as or better than both organic and conventional cow’s milk in all 12 of the environmental impacts examined, including land use, acidification and eutrophication potentials, ecotoxicity, and ecosystem damage. Soymilk scored similarly or better than cow’s milk for most of the same metrics [68••]. As demonstrated by variations in water use and scarcity, many of these environmental impacts are specific to the regions where animals, feed crops, and plant-based milk raw materials are produced. Individual studies often represent production in a specific region and thus may not be generalizable. Further research is needed to examine additional environmental impacts for other plant-based milks (e.g., pea, coconut, cashew), especially more peer-reviewed studies that assess multiple environmental impacts and dairy alternatives at once using the same methods, as encouraged by Röös et al. [81•].

## Conclusion

Retail sales of plant-based milks are increasing and shifting among product forms (e.g., almond milk overtaking soymilk), although plant-based milks still represent a small percentage of total US milk sales (Table 1). An incremental shift toward more plant-based milks could reduce food-related GHGe and, in most cases, lower water footprints. Different plant-based milks also vary greatly in their nutritional properties, and the potential long-term health implications of switching from dairy to plant-based milks are not well-studied.

Nutritional consequences of the recent shifts in dairy consumption are difficult to fully evaluate. Most plant-based milks cannot completely replace the nutritional quality of cow’s milk [48, 82]. Generally, a complete shift from dairy to plant-based milk would result in lower intakes of protein, phosphorous, and choline, as well as vitamin B12 and calcium, if the plant-based products are not fortified. Shifting within plant-based milk products would also have nutritional impacts. For example, pea milk could provide seven grams more protein per serving than almond milk.

More research is needed to assess the safety and nutritional value of using milk alternatives for growing children, as their nutrient requirements differ from adults. There is a potential risk of deficiencies of calcium, zinc, iodine, riboflavin, vitamin B12, and some essential amino acids in young children who are fed exclusively non-dairy milks [82]. Currently, the American Academy of Pediatrics recognizes only soy-based formulas or milks as safe and healthy for under 24 months of age, but there are many different plant-based products available for parents to choose. Accurately describing the differences between cow and plant-based milks will better prepare nutritionists, doctors, and public health officials to educate consumers who face many choices in the grocery store but little in the way of objective information.

Along with nutrient differences, there are environmental trade-offs related to particular milk types. While almond milk production results in less than half of the GHGe compared to a liter of cow’s milk, its water footprint can be substantially higher, especially if comparing almond milk produced in California to cow’s milk produced in a less water-scarce region. The more recent growth of oat milk is promising for lower GHGe and water footprints, but the protein content falls short of both soy and cow’s milk. Pea milk, though not yet widely available in markets, has lower GHGe and water footprints than dairy and most plant-based milks, and it provides a similar amount of protein as dairy milk.

To guide consumer purchasing and better inform policy makers, more research is needed to understand demographic and social characteristics, nutritional status, and intentions behind purchases of populations choosing to abstain from dairy milk, whether they are based on lactose intolerance, health or environmental concerns, advertising claims, price, preference, or other. Additionally, it would be useful to know what types of plant-based milks or other beverages they are substituting, if any. The decline in dairy milk sales is not explained in entirety by the growth in plant-based milk sales [3]. There is some thought that the increased availability of sweetened beverages across food environments is related to this decline, but the evidence is lacking [83].

Different purchasing patterns for dairy and plant-based milks impact affordability especially for financially constrained households. Many plant-based milks are significantly more expensive than dairy; however, for those with lactose intolerance, they may be more affordable than lactose-free milk, which may cost 50% more than milk. Yet, choosing oat or almond instead of lactose-free milk would provide 70 or 87% less protein, respectively. Manufacturers and policymakers can promote efforts to make nutritious, sustainable lactose-free and plant-based milks more affordable and available to consumers and develop standards for fortification to ensure plant-based milks are more similar nutritionally to cow’s milk.

This review reveals the need for more research in the international context as well. Are the findings of this study similar or different to consumption habits in Europe and other high-income nations? Have these new food products and trends influenced lower- and middle-income countries, particularly in Africa and Asia?

Finally, health professionals, environmentalists, economists, and those working toward a more healthy and sustainable food system should closely follow the rapidly changing market and consumer preferences for plant-based milks. As the market evolves, it is important to promote affordable products with adequate protein and micronutrients to maintain nutritional adequacy as well as those with lower ecological footprints.

## Supplementary Information

Below is the link to the electronic supplementary material.

Supplementary file1 (DOCX 17 KB)

Supplementary file2 (XLSX 69 KB)

## Data Availability

Data described in the manuscript are available in Supplementary Data Tables S1 through S9; analytic code will be made available upon request
