# Supplementary material for: Dairy and Plant-Based Milks: Implications for Nutrition and Planetary Health
Source: Curr Environ Health Rep. 2023 Jun 10;10(3):291–302. doi: 10.1007/s40572-023-00400-z (PMC10504201; doi:10.1007/s40572-023-00400-z)
Supplement: Supplementary file 1 — Supplementary file1 (DOCX 17 KB) [file 40572_2023_400_MOESM1_ESM.docx]

Additional methods for review of literature on the environmental impacts of dairy and plant-based milk alternatives

**Search strategy**

As discussed in the manuscript, literature was compiled on the greenhouse gas emissions (GHGe) and water use associated with the production of cow’s milk and plant-based milk alternatives. Environmental impact literature on dairy alternatives was compiled non-systematically through academic databases and Google Scholar searches. While there have been numerous life cycle assessments (LCAs) of cows milk, our inclusion criteria was limited to papers that included at least one plant-based milk alternative.

When reviewing four existing peer-reviewed studies that provided environmental impact data for dairy alternatives (Ercin et al., 2012; Grant & Hicks, 2018; Poore & Nemecek, 2018; Winans et al., 2020), as well as other LCA reviews (Clune et al., 2016), we found data cited from reports, conference presentations, and other gray literature. Given the novelty of the field, and limitations of existing peer-reviewed data—described more thoroughly by Röös et al. (2018)—we opted to include values reported in gray literature. In cases where we identified a peer-reviewed study that cited (and did not transform/modify) data from another primary source such as a report, we referred to and only cited the primary source (Table S1). We performed an additional search in Google Scholar to identify other gray literature and research published up to August 2022. Through this search process, several news articles were found which relied on unpublished additional calculations from Poore & Nemecek (2018) on the environmental footprints associated with several dairy alternatives (e.g., almond milk, oat milk, rice milk); these authors were contacted and their additional data was incorporated. One limitation of the decision to include non-peer reviewed research is that several of the LCA reports were funded or commissioned by companies selling dairy alternatives.

Grant & Hicks (2018) report water use, but the type of water use (e.g., water consumption v. withdrawals) is not indicated and the authors were unable to clarify this upon contacting them, thus we did not include their water use results in our review.

**GWP metric standardization**

Results for GHGe were standardized to reflect cradle to processor gate wherever possible. Thus GHGe associated with transportation from factory to wholesalers, retail, and consumers; retail (e.g., refrigeration); and consumer use (e.g., refrigeration, waste) were excluded unless they were unable to be disaggregated from earlier supply chain stages. The analysis also excludes GHGe associated with food losses and waste across the supply chain, as nearly all LCAs of dairy alternatives examined excluded such estimates. See the “note” column in Table S2 for details about whether and how footprints were disaggregated. If the time horizon associated with the global warming potential (GWP) was not reported, we assumed it was calculated based on 100 years (i.e., GWP 100).

**Water use metric standardization**

When standardizing footprints from a mass- to volume-based factor (e.g., from kg milk to L milk), the density of cow’s milk was used for all dairy alternatives (1 kg milk = 0.97 L milk). For the purposes of comparing plant milks to cow milk, for the two studies that did not provide a water use estimate for cow’s milk (Table S5), we compared the study values to the cow milk estimate from Mekonnen & Hoekstra (2010).

**Packaging**

Dairy alternatives may be packaged in several ways, including refrigerated types (plastic or liquid packaging board carton) or shelf stable, aseptic varieties. The existing literature was inconsistent in terms of which packaging types were assessed; thus, all packaging varieties were considered for this analysis.

**References**

Clune, S., Crossin, E., & Verghese, K. (2017). Systematic review of greenhouse gas emissions for different fresh food categories. *Journal of Cleaner Production*, *140*, 766-783.

Ercin, E., Aldaya, M., & Hoekstra, A. (2012). The water footprint of soy milk and soy burger and equivalent animal products. Ecoligical Indicators, 18, 392-402.

Grant, C. A., & Hicks, A. L. (2018). Comparative life cycle assessment of milk and plant-based alternatives. Environmental Engineering Science, 35(11), 1235-1247.

Mekonnen, M., & Hoekstra, A. Y. (2010). The green, blue and grey water footprint of farm animals and animal products. Volume 1: Main Report.

Poore, J., & Nemecek, T. (2018). Reducing food’s environmental impacts through producers and consumers. Science, 360(6392), 987-992.

Röös, E., Garnett, T., Watz, V., Sjörs, C. (2018). *The role of dairy and plant based dairy alternatives in sustainable diets.* SLU Future Food Reports 3. <https://pub.epsilon.slu.se/16016/1/roos_e_et_al_190304.pdf>

Winans, K. S., Macadam-Somer, I., Kendall, A., Geyer, R., & Marvinney, E. (2020). Life cycle assessment of California unsweetened almond milk. The International Journal of Life Cycle Assessment, 25(3), 577-587.
